# Multi-omics analysis reveals indicator features of microbe-host interactions during *Candida albicans* colonization and subsequent infection

**DOI:** 10.3389/fmicb.2024.1476429

**Published:** 2024-11-27

**Authors:** Huan Zhang, Daoyuan Song, Qiulin Luo, Jiangkun Yu, Yingpu Wei, Di Chen, Guangjuan Wu, Zhi Zhang, Zhao Li, Hongchao Jiang, Jingquan Gan, Deyao Deng, Hui Li, Wenli Yuan

**Affiliations:** ^1^Department of Clinical Laboratory, The Affiliated Hospital of Yunnan University (The Second People's Hospital of Yunnan Province), Kunming, China; ^2^Department of Neurology, The Affiliated Hospital of Yunnan University (The Second People's Hospital of Yunnan Province), Kunming, China; ^3^State Key Laboratory for Conservation and Utilization of Bio-Resources in Yunnan, School of Life Sciences, Yunnan University, Kunming, Yunnan, China; ^4^The Kunming Children’s Hospital, Kunming, China; ^5^Department of Infectious Disease, The Affiliated Hospital of Yunnan University (The Second People's Hospital of Yunnan Province), Kunming, China

**Keywords:** *Candida albicans*, intestinal colonization, invasive infection, multi-omics analysis, programmed death-1

## Abstract

**Introduction:**

*Candida albicans* gastrointestinal (GI) colonization is crucial for the onset of invasive disease. This research encompassed 31 patients diagnosed with *Candida* spp. bloodstream infections during their admission to a university hospital in China.

**Methods:**

We explored risk factors associated with *C. albicans* GI colonization and ensuing translocated infection. Animal models were established via gavage with clinical isolates of *C. albicans* to induce GI tract colonization and subsequent kidney translocation infection. Our analysis is focused on 16S rRNA gene sequencing, metabolomics of colon contents, and transcriptomics of colon tissues, examining the intestinal barrier, inflammatory responses, and immune cell infiltration.

**Results:**

This study observed that down-regulation of programmed cell death 1 (PD-1) in colon tissues is likely linked to the progression from *C. albicans* colonization to translocated infection. Notably, reductions in *Dubosiella* abundance and Short-chain fatty acids (SCFA) levels, coupled with increases in *Mucispirillum* and D-erythro-imidazolylglycerol phosphate, were indicator features during the advancement to translocated invasive infection in hosts with rectal colonization by *C. albicans* and lower serum protein levels.

**Conclusion:**

Given the similarity in intestinal bacterial communities and metabolome profiles, antifungal treatment may not be necessary for patients with nonpathogenic *C. albicans* colonization. The reduced expression of PD-1 in colon tissues may contribute to the transition from colonized *C. albicans* to subsequent translocated infection. The indicator features of decreased Dubosiella abundance and SCFA levels, coupled with increased Mucispirillum and D-erythro-imidazolylglycerol phosphate, are likely linked to the development of translocated invasive infection in hosts colonized rectally by *C. albicans* with lower serum protein levels.

**Importance:**

*Candida albicans* invasive infections pose a significant challenge to contemporary medicine, with mortality rates from such fungal infections remaining high despite antifungal treatment. Gastrointestinal colonization by potential pathogens is a critical precursor to the development of translocated infections. Consequently, there is an increasing demand to identify clinical risk factors, multi-omics profiles, and key indicators to prevent the progression to translocated invasive infections in patients colonized rectally by *C. albicans*.

## 1 Introduction

*Candida* is a diverse genus of fungi consisting of over 200 species. One of the most prevalent is *Candida albicans*, a commensal fungus colonizes in the gastrointestinal (GI) tract without causing infection (Mesquida et al., 2022; Alonso-Monge et al., 2021; Mesquida et al., 2023). To distinguish lifestyle of *C. albicans* in GI tract as commensal or pathogenic colonization has great clinical importance (Prasad and Tippana, 2023). The most distinct features of *C. albicans* pathogenic colonization is hostile takeover of the host niche, by means of disturbing and remodeling gastrointestinal microbiota (Liang et al., 2024; Yan et al., 2024). Hosts with pathogenic colonization exhibit increased abundance of *C. albicans* but altered bacterial diversity, attributable to linear alterations in specific bacterial loads and/or their metabolites (Peroumal et al., 2022). Furthermore, *C. albicans* impacts on early microbiome community construction by inhibiting various dominant genera of intestinal bacteria (Zeise et al., 2021). In animal models, an antagonistic relationship was observed between *C. albicans* and *Lactobacillus johnsonii*, along with a positive correlation between *C. albicans* colonization and *E. faecalis* levels (Vazquez-Munoz et al., 2023). Highlighting, the interactions of *C. albicans* with gastrointestinal microbiota contribute to one’s growth and virulence (Li et al., 2022; Kumamoto et al., 2020). Based on this fact, it is crucial for clinical microbiology laboratories to identify both the interacting bacterial communities and the complex metabolic niches involved.

*Candida albicans* can lead to life-threatening infections, with an increased mortality rate in immunocompromised or in microbial dysbiosis patients, even with antifungal treatment (Lass-Flörl and Steixner, 2023). It is crucial to note that more than 60% of global cases of invasive candidiasis are caused by *C. albicans*, originating from pathogenic colonization in the gastrointestinal tract. *C. albicans* is known to alternate among multiple morphologies, including the cellular and hyphal forms (Ost et al., 2021). The cellular form is much more abundant in the colon of gnotobiotic mice mono-colonized with *C. albicans*, and its transition to the hyphal form is associated with greater virulence. Thus, the cellular form is thought to be better suited for invasive infections (Witchley et al., 2019). *C. albicans* may compromise the gut barrier via multiple mechanisms (Kadosh et al., 2016; Sprague et al., 2024). *C. albicans* therefore is capable of invading intestinal tissue and facilitating subsequential translocation of candidiasis. Regarding to potential life-threatening outcome of *C. albicans* pathogenic colonization, early predication is urgently required in clinical practice. Recent research (Zeise et al., 2021; Atanasov et al., 2023) has identified that variations in lactic acid bacteria levels within the GI microbiome could serve as potential biomarkers for predicting *C. albicans* colonization, dissemination, and subsequent infection. Therefore, we hypothesize that certain bacteria and/or metabolites probably contributed to *C. albicans* colonization, translocation and invasion.

We identified the independent risk factors for the *C. albicans* fecal colonization and subsequently translocated bloodstream infection in patients from a university hospital in China. Furthermore, we conducted animal experiments by using *C. albicans* clinical isolates which were inoculate into GI tracts in order to explore pathogenesis and etiology, especially for systematic candidiasis. To determine such shift and potential candidates in biomarker for *C. albicans* pathogenic colonization, we employed a multi-omics approach to investigate microbiome variations, metabolome and host gene expression. Furthermore, the integrity of the intestinal barrier, inflammatory responses, and immune cell infiltration were extensively analyzed by using colon tissues from animal models exhibiting *C. albicans* colonization and subsequent systematic infection. Our objective was to identify risk factors and intestinal biomarkers for subsequent infections in hosts colonized by *C. albicans*, aiming to facilitate early diagnosis and treatment.

## 2 Materials and methods

### 2.1 Clinical study design

This study was conducted in a tertiary care hospital in Yunnan Province, China, from January 1, 2019, to July 31, 2023. Throughout this timeframe, inpatients with *C. albicans* fecal colonization were selected. The inclusion criteria for participants necessitated three consecutive quantitative fecal fungal cultures yielding more than 10^4 cfu/g, with the isolates identified as *C. albicans*. Two participants later exhibited *C. albicans* in blood, which clinicians deemed as bloodstream infections. Additionally, individuals diagnosed with *Candida* spp. bloodstream infections within the same timeframe were recruited. Eligibility for this cohort was based on meeting the diagnostic criteria for *Candida* spp. bloodstream infections, having comprehensive clinical records, and being over 18 years of age.

### 2.2 Medical records collection and analysis

Clinical records of patients in our study were gathered through the digital hospital database system. Despite gender and age, the clinical variables analyzed included details on hormone therapy, such as glucocorticoid treatments with prednisone, methylprednisolone, dexamethasone, and hydrocortisone. The underlying diseases identified included hypertension, diabetes mellitus, cardiovascular diseases, chronic kidney disease, hepatic insufficiency, heart diseases, and chronic obstructive pulmonary disease (COPD). Hypoproteinemia was defined as having serum total protein levels below 60 g/L. The analysis of prior antibiotic therapy included treatments with carbapenems, quinolones, *β*-lactams, aminoglycosides, tetracyclines, enzyme inhibitor antibiotics, and macrolide antibiotics. Additionally, data on ICU admissions, mechanical ventilation use, and mortality rates among infected patients were collected.

### 2.3 Animal experiments

Specific-pathogen-free (SPF) C57BL/6J female mice, aged 6–8 weeks, were utilized in the experiment. Mice underwent a 5-day acclimatization period and randomly allocated into three groups: the control group, the *C. albicans* colonization group (colonization), and the *C. albicans* translocation infection group (translocation), with six mice per group. To prepare the *C. albicans* inoculation, a concentration of 1 × 10^8 CFU/mL was achieved. To induce gastrointestinal colonization, immunosuppressed C57BL/6 J mice, treated intravenously with 150 mg/kg of cyclophosphamide and levofloxacin supplementation at a dosage of 10 mg/kg. Following a 14-days treatment period, these mice were orally administered *C. albicans*. 7 days after 14-days treatment of cyclophosphamide, levofloxacin and oral gavage *C. albicans*. Upon successful establishment of the colonization model, the mice were separated into two groups, with six mice in each: one group for studying *C. albicans* GI colonization, the other group mice were subjected to additional challenges of oral gavage with *C. albicans* and cyclophosphamide injection for other 7 days for establishing translocated infection model. By approximately day 28, models of *C. albicans* translocated infection had been established. The mice were then sacrificed, and samples from the colon and kidneys were collected for analysis. Quantitative cultures and RT-PCR were performed to assess *C. albicans* loads in colonial contents and kidneys. Furthermore, fungal immunofluorescence chromogenic staining was conducted on kidney tissue samples. Supplementary Figure 1 illustrates significant *C. albicans* growth and elevated 18S expression in colon contents, confirming the successful establishment of the *C. albicans* gastrointestinal (GI) colonization animal models. Additionally, the presence of *C. albicans*, pronounced 18S expression, and immunohistochemical staining in kidney tissues verified the successful creation of *C. albicans* translocated infection models.

### 2.4 16S rRNA gene sequence

Colonial contents of mice were collected, promptly frozen in liquid nitrogen, and transported to Personalbio Technology for processing and Illumina high-throughput sequencing. Sequencing results were segregated into libraries and samples using index and barcode information, with barcode sequences removed. QIIME2 dada2 was used for sequence denoising and amplicon sequence variants (ASVs) generate. Alpha and Beta diversity analyses, as well as bacterial community composition analysis, were conducted after subsampled at an even depth of 10,000 reads for each sample.

### 2.5 Short-chain fatty acid extraction and gas chromatography–mass spectrometry

Colonial contents of mice were collected, promptly frozen in liquid nitrogen, and transported to Personalbio Technology for processing for Short-chain fatty acids (SCFA). The GC–MS system consisted of Agilent 7890A/5975C (Agilent, USA). MSD Chem Station (Agilent, USA) software was used to extract chromatographic peak area and retention time.

### 2.6 Ultraperformance liquid chromatography–tandem mass spectrometry assay for nontargeted metabolomics

Colonial contents of mice were collected, promptly frozen in liquid nitrogen, and transported to Personalbio Technology for processing and then analyzed using ultra-high performance liquid chromatography–tandem mass spectrometry (UHPLC–MS/MS). Significantly different metabolites were initially selected on the basis of the variable important in projection (VIP) and Student’s t-test *p*-values. Metabolites with VIP ≥ 1 and *p* < 0.05 were generally considered significantly different. Finally, through metabolic pathway annotation in the KEGG database,^1^ the pathways in which differential metabolites were involved were obtained, and KEGG pathways were considered significantly enriched for *p* < 0.05.

### 2.7 Transcriptomic analysis

Colonial contents of mice were collected, promptly frozen in liquid nitrogen, and transported to Personalbio Technology for processing, DESeq2 was used to perform differential gene expression analysis between sample groups. The differential expression fold (foldchange, FC) of each gene was calculated, and the differentially expressed genes (DEGs) were screened by using |log2 FC| ≥ 1and *p* < 0.05 as the standard. KEGG pathway enrichment analysis was performed on the DEGs.

### 2.8 Reverse-transcription quantitative polymerase chain reaction (RT-PCR)

Total RNA was extracted using TRIzol reagent (Thermo, USA) according to the manufacturer’s protocol. RNA was then reverse transcribed with the Revert First Strand cDNA Synthesis Kit (Thermo, USA) according to the manufacturer’s instructions. RT-PCR was performed using ABI-7500 (Applied Biosystems, USA). RT-PCR was performed in a total volume of 10 μL with the following amplification conditions: 95°C for 3 min and then 95°C for 10 s and 60°C for 30 s, for 40 cycles. The melt curve was completed with the following cycle conditions: 95°C for 10 s and 65°C for 10 s with an increase of 0.5°C per cycle up to 95°C. RT-PCR were performed to assess *C. albicans* loads in colonial contents and kidneys. Colonial contents and kidneys were tested three times. The 2^−ΔΔCt^ approach was used to calculate the relative expression levels of differential genes among the groups.

### 2.9 ELISA

The concentrations of cytokines in colon tissue were measured by ELISA kits (CusaBio, Wuhan, China). Colonic IL-6 and IL-10 quantitative ELISA kits were used according to the instructions. The concentrations of occludin in colon tissue were measured by ELISA kits (RUIXIN BIOTECH, Quanzhou, China).

### 2.10 Hematoxylin and eosin (HE) and immunohistochemical staining

Paraffin-fixed tissue samples were sliced into 5 mm thick sections and stained with HE and immunohistochemical (CD68 (1:200), CD19 (1:200), CD56 (1:200), MPO). Immunohistochemistry results were observed using a fluorescence microscope (Olympus). Histological changes were assessed by two blinded experienced pathologists at the same time using a previously described scoring system, and the average score was taken.

### 2.11 Statistical analysis

GraphPad Prism 7.0 and SPSS 21.0 were used to analyze data. Univariate analyses were performed separately for each of the variables. Categorical variables were compared using a chi-square test. The Odds ratios (ORs) and their corresponding 95% confidence intervals (CIs) were calculated. Variables with *p* < 0.05 on univariate analysis were evaluated as potential covariates in a stepwise multivariate logistic regression model. Continuous variables were compared using Student’s t-test (normally distributed variables) and Wilcoxon rank-sum test (non-normally distributed variables) as appropriate.

## 3 Results

### 3.1 The independent risk factors for the *Candida albicans* fecal colonization and subsequently bloodstream infection in patients

In cross-sectional study of 30 participants, we evaluated the factors contributing to gastrointestinal colonization by *C. albicans*. Logistic regression analysis (*p* < 0.05) reveals significant associations with hormone therapy, hypoproteinemia, and the presence of three or more underlying diseases. Further, hormone therapy and hypoproteinemia were identified as independent risk factors for *C. albicans* colonization in the gastrointestinal tract (Table 1) 0.30 patients were identified as rectal carriers of *C. albicans*, of which only two developed a bloodstream infection with *C. albicans*, as confirmed by positive blood cultures and clinical interpretation. Subsequently, a cross-sectional study was conducted with 31 patients diagnosed with *Candida* spp. bloodstream infections during their hospital stay. The cohort included 18 males (58.1%) and 13 females (41.9%), with an average age of 62 years (ranging from 38 to 91 years). A notable mortality rate of 32.3% was observed among these patients. Furthermore, using the Chi-square test (*p* < 0.05), patients with *Candida* spp. bloodstream infections were found to have longer hospital stays and reduced serum albumin levels. Additionally, these patients were more likely to have had extensive prior antibiotic therapy (≥3 courses) (*p* < 0.001) and to have undergone mechanical ventilation compared to the *C. albicans* rectal carriers. No significant differences in gender or age were noted between the two groups. Binary logistic regression analysis indicated that hypoproteinemia was a significant independent factor affecting the likelihood of developing fungal bloodstream infections (*p* = 0.015) (Table 2).

**Table 1:** Logistic regression analysis of clinical variables associated with *C. albicans* fecal colonization in patients.

Clinical factors	Univariate analysis		Multivariate analysis	
	OR (95%CI)	*p* value	OR (95%CI)	*p* value
Hormone therapy	0.043 (0.005–0.376)	0.004	0.049 (0.006–0.435)	0.007
Underlying disease ≥ 3	0.263 (0.074–0.936)	0.039	–	–
Hypoproteinemia	0.009 (0.001–0.084)	<0.001	0.015 (0.001–0.294)	0.006

**Table 2:** Logistic regression analysis of clinical variables associated with *Candida* spp. bloodstream infection in patients.

Clinical variable	Univariate analysis		Multivariate analysis	
	χ2value	*p* value	Hazard ratio (95% CI)	*p* value
Age ≥ 60	0.042	0.837		
Gender	0.177	0.674		
Prior antibiotic therapy ≥ 3	27.64	<0.001		
Underlying disease ≥ 3	2.765	0.096		
Hypoproteinemia	19.042	<0.001	14.769 (1.691–129.004)	0.015
Mechanical ventilation	14.385	<0.001		

### 3.2 *Candida albicans* colonization and translocation infection influenced the diversity and the composition of bacterial community

**Figure 1:**
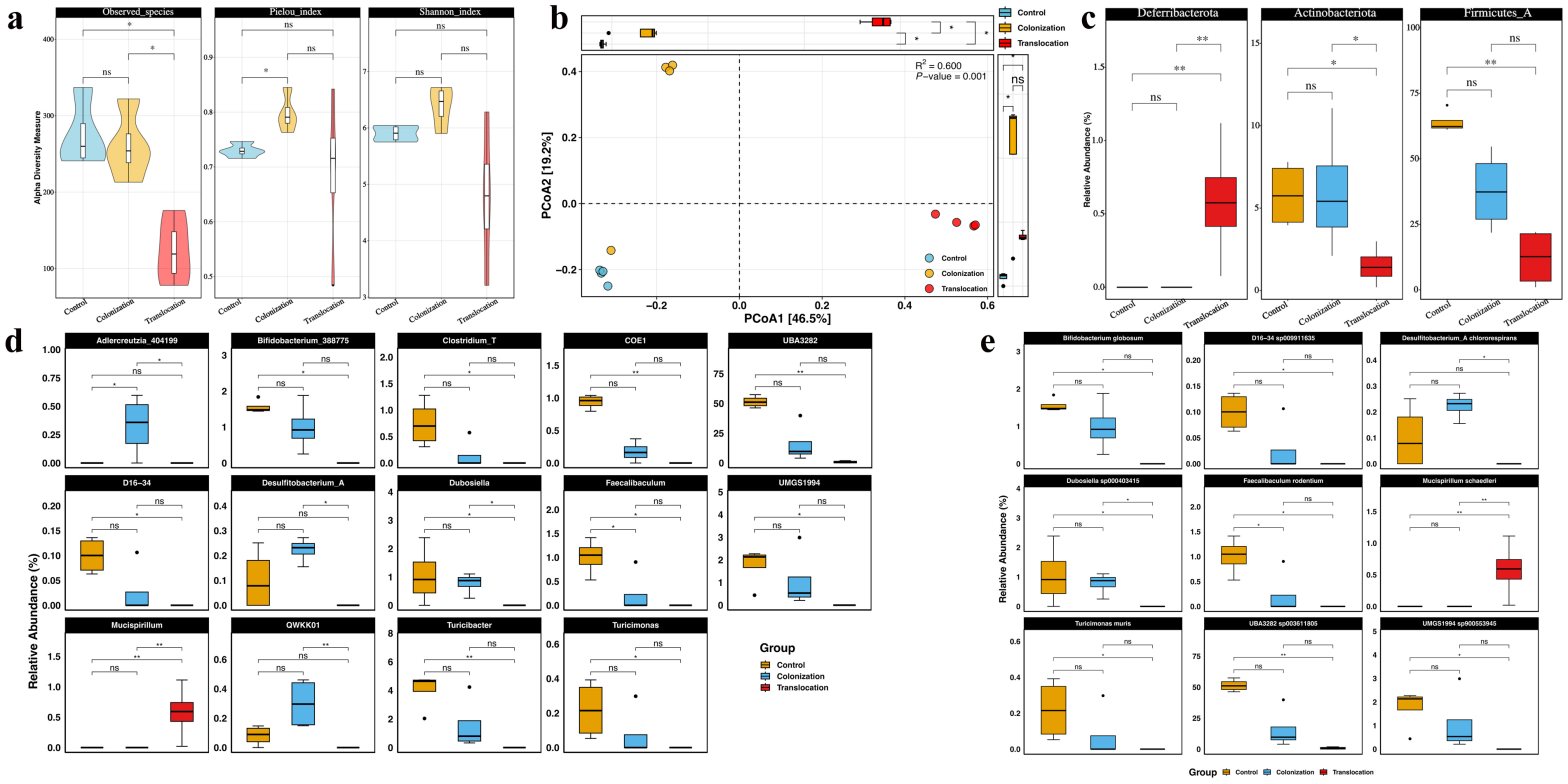
*C. albicans* colonization and translocation infection influence the diversity and the composition of microbiota. To assess the impact of *C. albicans* GI colonization and translocation infection on the diversity and composition of bacterial community, 16S rRNA gene amplicon sequencing was performed on the colonic content of the three groups. **(A)** Alpha diversity indicated by observed species, Shannon_index, and Pielou_index. **(B)** PCoA of beta diversity. **(C)** Statistical analysis results of bacterial relative abundance at the phylum level. **(D)** Statistical analysis results of bacterial relative abundance at the genus level. **(E)** Statistical analysis results of bacterial relative abundance at the species level (*p < 0.05, **p < 0.001).

To evaluate the effects of *C. albicans* gastrointestinal colonization and translocation infection on the diversity and composition of the bacterial community, 16S rRNA gene amplicon sequencing was conducted on colonic content from the three groups. Figure 1A illustrates that alpha diversity was assessed using the Observed Species, Pielou, and Shannon indices. Notably, the Observed Species index significantly decreased following *C. albicans* translocation infection compared to the GI colonization group, whereas the Pielou and Shannon indices did not exhibit statistically significant differences between these groups. Furthermore, alpha diversity remained largely unchanged under *C. albicans* GI colonization, with the exception of an increase in the Pielou index. Beta diversity, analyzed through Principal Coordinate Analysis (PCoA) based on Bray-Curtis distances, revealed distinct gut microbial community structures among the three groups (PERMANOVA: *R*^2^ = 0.6, *p* = 0.001) as depicted in Figure 1B.

Differences in the relative abundance of microorganisms among the three groups were analyzed. *Firmicutes* and *Bacteroidota* emerged as the most prevalent phyla in all samples from the three groups. Significantly, *Deferribacterota*’s relative abundance was markedly increased in the *C. albicans* translocation infection group compared to the colonization groups, whereas *Actinobacteriota*’s presence significantly diminished, as illustrated in Figure 1C. However, the relative abundances of *Deferribacterota* and *Actinobacteriota* in the *C. albicans* colonization group did not significantly differ from those in the control group.

Detailed analysis presented in Figures 1D,E shows significant reductions in the relative abundance of *Desulfitobacterium*_A and *Dubosiella* at both genus and species levels in the *C. albicans* translocated infection group compared to mice with GI colonization. Interestingly, a majority of bacterial taxa maintained comparable levels to control mice in response to *C. albicans* GI colonization. Most notably, the abundance of *Adlercreutzia* and *Mucispirillum* was markedly increased within their respective bacterial communities in models challenged with *C. albicans* GI colonization, regardless of the presence of translocated infection.

### 3.3 Metabolomics analysis indicated that *Candida albicans* GI colonization and translocated infection possessed versatile metabolites

**Figure 2:**
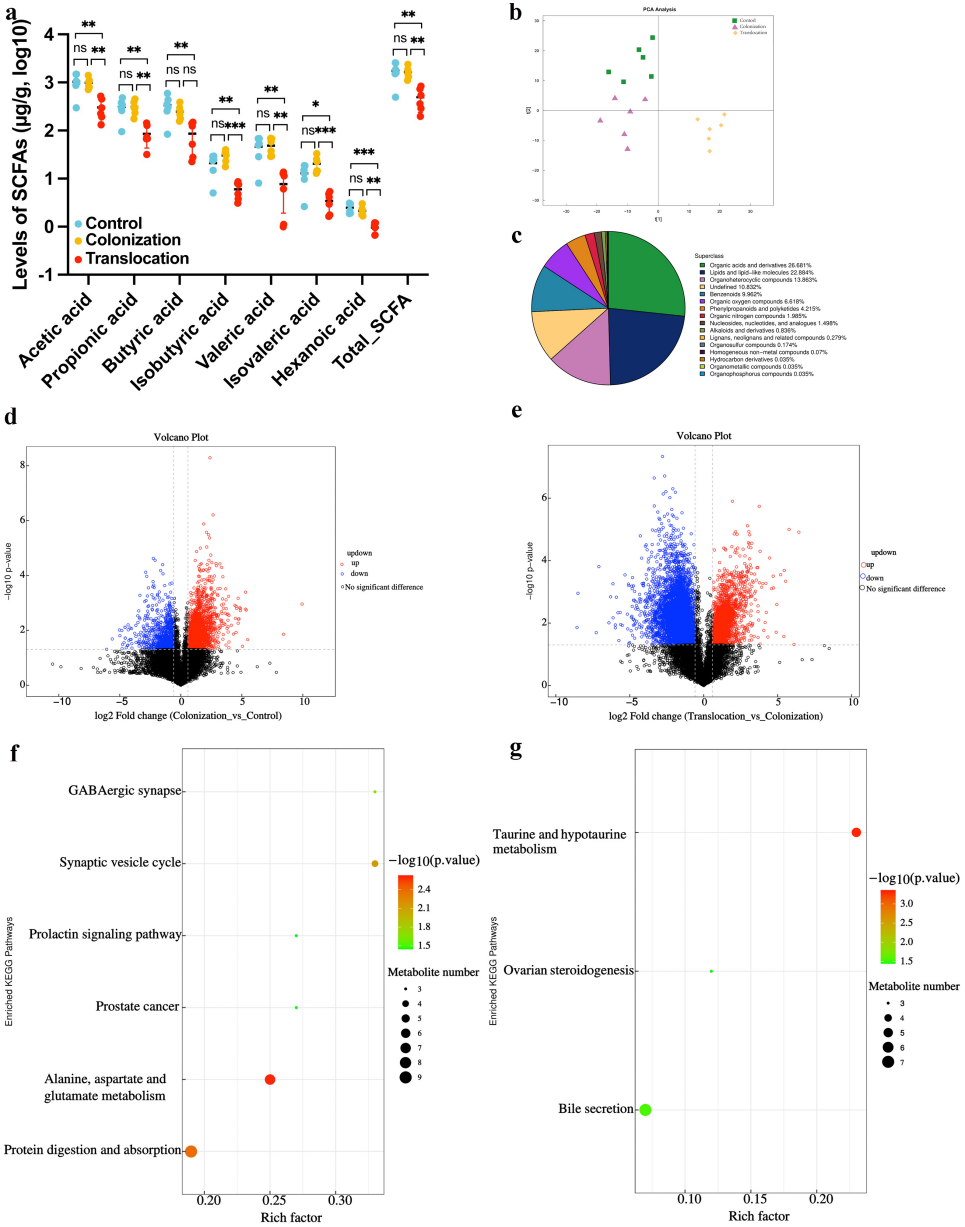
Metabolome analysis of colonial contents. To investigate whether *C. albicans* colonization and translocated infection induce metabolic alteration, we analyzed non-targeted metabolomics and SCFAs levels in colonial contents collected from models. **(A)** Results of statistical analysis of short-chain fatty acid metabolites. **(B)** PCA between the control group, *C. albicans* GI colonization group and *C. albicans* subsequently translocated infection group. **(C)** Classification of differential metabolites in the HMDB database. **(D)** Volcano plot of differential metabolites between the control group and *C. albicans* colonization group. Red dots (up) represent significantly upregulated metabolites (*p* < 0.05, FC ≥ 2); blue dots (down) represent significantly downregulated metabolites (*p* < 0.05, FC ≤ 0.5); and black dots (no) represent insignificant metabolites. **(E)** Volcano plot of differential metabolites between the *C. albicans* colonization group and *C. albicans* subsequently translocated infection group. Red dots (up) represent significantly upregulated metabolites (*p* < 0.05, FC ≥ 2); blue dots (down) represent significantly downregulated metabolites (*p* < 0.05, FC ≤ 0.5); and black dots (no) represent insignificant metabolites. **(F)** Bubble map of metabolites categorized through KEGG pathway enrichment analysis when compared control group with *C. albicans* colonization group. **(G)** Bubble map of metabolites categorized through KEGG pathway enrichment analysis when compared *C. albicans* colonization group and *C. albicans* subsequently translocated infection group (*p < 0.05, **p < 0.01, ***p < 0.001).

SCFAs are crucial metabolites predominantly produced by the gut microbiota. In mice with *C. albicans* gastrointestinal (GI) colonization followed by translocated infection, the concentrations of acetic acid, propionic acid, butyric acid, isobutyric acid, valeric acid, isovaleric acid, and hexanoic acid in colon contents were significantly reduced. Conversely, no notable differences in SCFA levels were detected in the colon contents of mice with *C. albicans* GI colonization when compared to the control group, as depicted in Figure 2A.

To determine if *C. albicans* colonization and translocated infection trigger metabolic disturbances in feces, we utilized UPLC-MS/MS for non-targeted metabolomic analysis of colon content samples from the models. This comprehensive metabolomic study identified a total of 2,871 metabolites, integrating both positive and negative ionization modes. As depicted in Figure 2B, two distinct metabolome profiles emerged from the analysis of the three groups, suggesting that *C. albicans* translocated infection may result in a unique intestinal metabolome pattern. Similar to the SCFA findings, the metabolome profile of the *C. albicans* GI colonization group closely matched that of the control group.

Statistical analysis revealed significant alterations in the abundance of 762 metabolites across the three groups. Specifically, organic acids and derivatives (26.681%), lipids and lipid-like molecules (22.884%), and organoheterocyclic compounds (13.863%) predominated among the differentially abundant metabolites (Figure 2C). Detailed subsequent analysis, as illustrated in Figures 2D,F, showed that 233 metabolites (152 up-regulated and 81 down-regulated) differed between the *C. albicans* GI colonization group and the control group (Figure 2D). These 233 altered metabolites in the colon content from the colonization group were significantly associated with six pathways: GABAergic synapse, synaptic vesicle cycle, prolactin signaling pathway, prostate cancer, alanine, aspartate and glutamate metabolism, and protein digestion and absorption (Figure 2F). Moreover, in the context of *C. albicans* translocated infection, 267 differential metabolites (124 up-regulated and 143 down-regulated) were identified in the *C. albicans* GI colonization mice (Figure 2E), predominantly affecting three pathways: taurine and hypotaurine metabolism, bile secretion, and ovarian steroid production (Figure 2G).

### 3.4 Transcriptomic profiling of the colonic tissue

**Figure 3:**
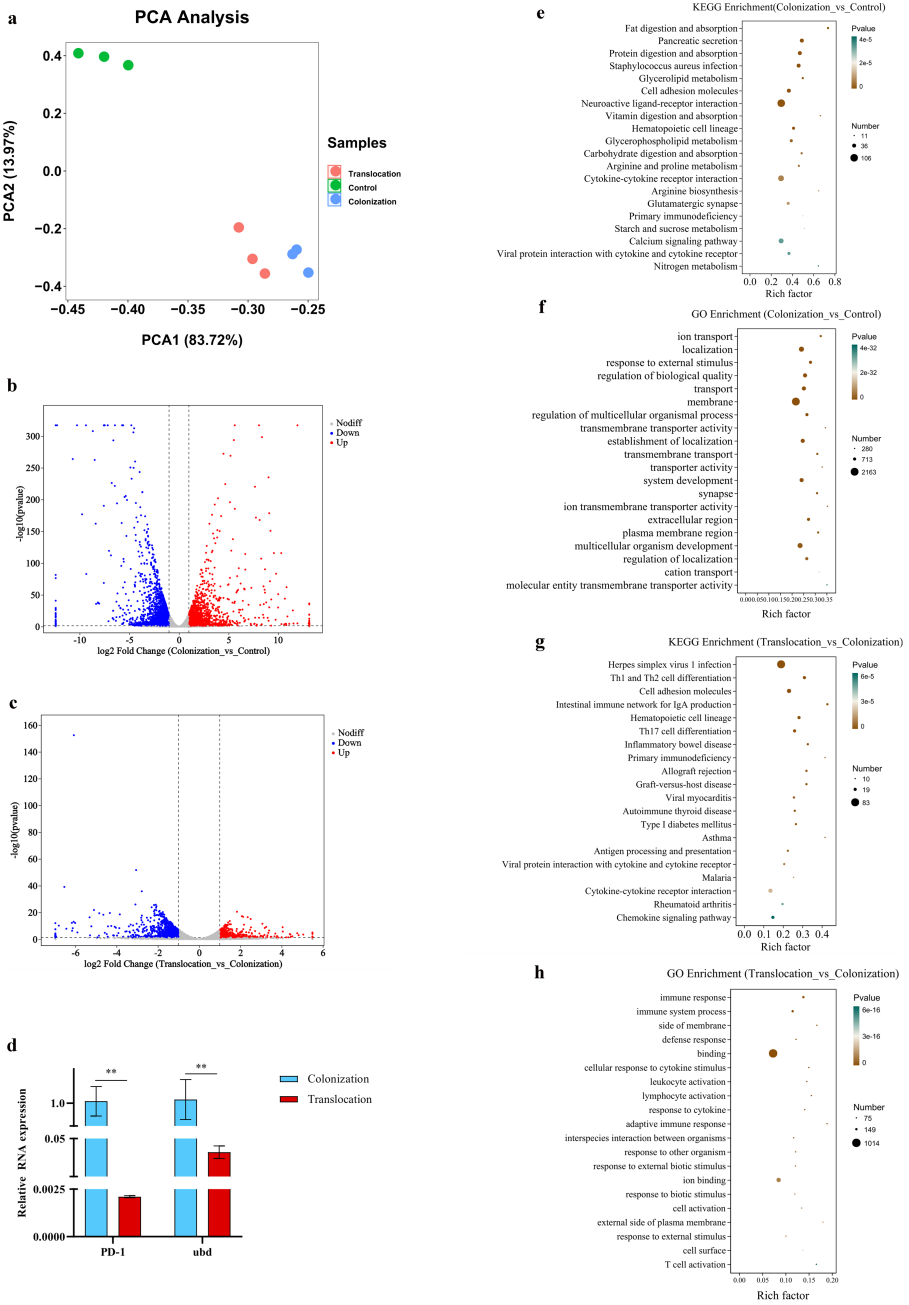
Transcriptome analysis. To investigate the host gene expression modulated microbial ecology as well as bacterial metabolites, which allowing colonized *C. albicans* to invade the intestinal tissue and subsequently translocated infection, transcriptomic analysis of colon samples was performed. **(A)** PCA between the control, *C. albicans* GI colonization and *C. albicans* subsequently translocated infection group. **(B)** Volcano plot of DEGs between the control group and *C. albicans* GI colonization group. Red dots (up) represent significantly upregulated DEGs (*p* < 0.05, FC ≥ 2); blue dots (down) represent significantly downregulated DEGs (*p* < 0.05, FC ≤ 0.5); and black dots (no) represent insignificant DEGs. **(C)** Volcano plot of DEGs between *C. albicans* GI colonization group and *C. albicans* subsequently translocated infection group. Red dots (up) represent significantly upregulated DEGs (*p* < 0.05, FC ≥ 2); blue dots (down) represent significantly downregulated DEGs (*p* < 0.05, FC ≤ 0.5); and black dots (no) represent insignificant DEGs. **(D–G)** Bubble chart of the KEGG or GO pathways (top 20) that DEGs significantly involved in. **(H)** Relative mRNA expressions of indicator genes in the colon tissues harvested from *C. albicans* GI colonization and subsequently translocated infection mice (**p < 0.001).

This study aimed to explore how host factors are influenced by the altered microbial ecology and bacterial metabolites, facilitating *C. albicans*’ invasion and subsequent translocation into the intestinal tissue. Transcriptomic analysis was conducted on colon samples from mouse models to achieve this objective. The RNA’s quantity and quality were meticulously evaluated for each sample. The experimental design included three samples each from the control group, the *C. albicans* gastrointestinal (GI) colonization group, and the group with subsequent *C. albicans* translocation infection. Principal component analysis, depicted in Figure 3A, revealed a clear distinction among these groups. The findings suggest that the changes in microbial ecology and bacterial metabolites in response to *C. albicans* colonization and translocation could significantly affect gene expression in the mice models.

The transcriptomic analysis revealed a total of 9,503 host genes were annotated, among which 523 genes were common across the control, *C. albicans* GI colonization, and translocation infection groups. In comparison to the control group, colonic tissues challenged with *C. albicans* GI colonization showed 1,668 genes significantly up-regulated and 2,227 genes down-regulated (Figure 3B). Conversely, the subsequent translocation infection resulted in 1,378 differentially expressed genes, with 504 up-regulated and 874 down-regulated, relative to the colonization group (Figure 3C). Detailed enrichment analyses of the differentially expressed genes (DEGs) across groups were conducted, as shown in Figure 3. KEGG pathway (Figures 3D–G) and GO analyses (not presented) were utilized to elucidate the pathway participation of these genes. Notably, several key cellular signaling pathways, including cytokine-cytokine receptor interaction, calcium signaling, Th17 cell differentiation, Th1 and Th2 cell differentiation, and the intestinal immune network for IgA production, were implicated in the experimental mice.

Additionally, we assessed the expression levels of the indicator genes, programmed cell death 1 (PD-1) and ubiquitin D (Ubd), using real-time quantitative PCR. Figure 3 illustrates a down-regulation of both PD-1 and Ubd in colon tissues from mice with *C. albicans* translocated infection, relative to GI colonization by *C. albicans*.

### 3.5 Multiomics analysis along the microbiome-metabolite-host axis in mice with *Candida albicans* colonization and subsequently translocation infection

**Figure 4:**
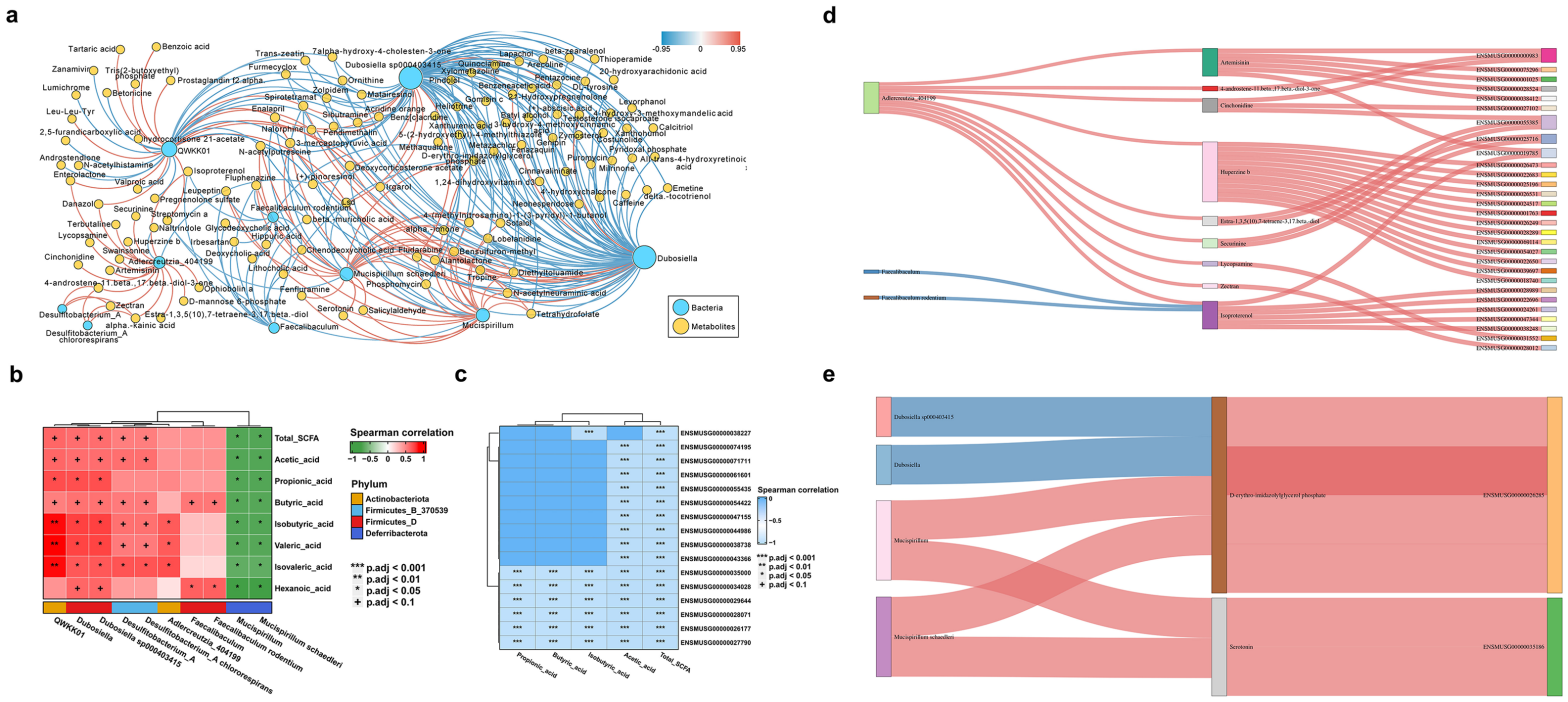
Multiomics analysis of mice with *C. albicans* colonization and translocation infection. To explored whether the altered microbial ecology as well as bacterial metabolites modulate the host gene expression to allowing *C. albicans* colonized in the intestinal turn to subsequently translocated infection. Spearman correlation analysis was conducted to calculate the possible correlation between all differential transcripts, metabolites and bacteria genera. **(A)** Overall co-occurrence network of bacterial indicator genera (blue labels) and differential non-target metabolites (yellow labels) during the development from *C. albicans* GI colonization group to *C. albicans* subsequently translocated invasive infection. Edges with pink and blue indicate significantly positive and negative correlations, respectively. The distribution of network degrees corresponds to node sizes. The SparCC method was used to perform correlation analysis, and the co-occurrence network was visualized by Cytoscape v3.9.1. **(B)** Heatmap showing significant microbial contributors to the SCFAs metabolites analyzed by the Pearson correlation. The value of the Pearson correlation coefficient was represented by the color label. The correlation coefficient R is shown in color. *R* > 0 indicates positive correlation, represented by red; *R* < 0 indicates negative correlation, represented by green. *p*-value reflects the significance level of correlation. *p*-value <0.05 is represented by *, *p*-value <0.01 is represented by **, and *p*-value <0.001 is represented by ***. **(C)** Heatmap of the correlation coefficient matrix between DEGs and SCFAs analyzed by the Pearson correlation. The value of the Pearson correlation coefficient was represented by the color label. The correlation coefficient R is shown in color. *R* < 0 indicates negative correlation, represented by blue. *p*-value reflects the significance level of correlation, and *p*-value <0.001 is represented by***. **(D,E)** Sankey diagram delineating all interaction links between indicator microbial genera, inferred metabolites, and host marker genes. When compared the control group and *C. albicans* GI colonization group, the analysis results was showed in panels **(D,E)** represent the analysis results between *C. albicans* GI colonization group and *C. albicans* subsequently translocated infection group. Metabolites were grouped by their classes in PubChem. Host genes were grouped by the enriched pathways and only genes in the top pathways (FDR *p* < 1e−8) are shown. Edges with pink and blue indicate significantly positive and negative correlations, respectively.

To assess the impact of gut microbiota changes due to *C. albicans* colonization on host metabolism, we examined the relationships between 10 indicator bacterial genera and 118 differentially expressed non-targeted metabolites in colon contents during *C. albicans* colonization and subsequent translocated infection. The co-occurrence network, generated through SparCC correlation analysis, revealed 93 positively and 184 negatively correlated pairs. This network underscores the intricate interactions between the microbiome and metabolome within the intestinal microbial community during *C. albicans* colonization (Figure 4A). Notably, *Dubosiella* exhibited the largest network degree and showed significant correlations with various bacteria and host metabolites. Additionally, our analysis identified associations between 10 distinct bacterial genera and SCFAs extracted from colon contents in both colonization and translocation infection groups (Figure 4B). In particular, *Dubosiella* and *Desulfitobacterium*_A demonstrated positive correlations with all SCFAs. However, the co-expression networks linking specific SCFAs (Isobutyric acid, Valeric acid, and Isovaleric acid) with *Mucispirillum schaedleri* showed negative associations.

A heatmap analysis was conducted to explore the correlations between differentially expressed genes and indicator SCFA metabolites in the *C. albicans* colonization group and the translocated infection models. Notably, changes in SCFAs, particularly acetic acid, influenced various genes in the host colon from colonization to invasive infection. Illustrated in Figure 4C, the heatmap reveals significant genes, ordered from top to bottom: homeobox A9 (Hoxa9), chloride channel accessory 4B (Clca4b), mercaptopyruvate sulfurtransferase (Mps), presynaptic cytomatrix protein (Pclo), MAF bZIP transcription factor (Maf), fatty acid binding protein 1 (Fabp1), cytochrome P450 family 4 subfamily x polypeptide 1 (Cyp4x1), thiosulfate sulfurtransferase (Tst), SH3 and multiple ankyrin repeat domains 1 (Shank1), olfactory receptor family 51 subfamily E member 2 (Or51e2), dipeptidylpeptidase 4 (Dpp4), CD226, pancreatic and duodenal homeobox 1 (Pdx1), leucine rich adaptor protein 1 (Lurap1), solute carrier family 11 member 1 (Slc11a1), and sucrase-isomaltase (Sis).

In this study, we delved into whether changes in microbial ecology and bacterial metabolites influence host gene expression, thereby facilitating the progression of *C. albicans* colonization in the intestinal tissue and subsequent translocated infection. Spearman correlation analysis was employed to assess potential correlations among differential transcripts, metabolites, and bacterial genera. Integrating indicator bacterial genera, the differential metabolome, and transcriptome data between the *C. albicans* GI colonization and the control group (Figure 4D), we also examined the correlations involving 3 bacterial genera, 9 metabolites, and 29 genes. Figure 4A reveals that the correlation analysis highlighted complex interactions between the microbiome and the host.

Integrating differential bacterial genera with indicator metabolome and transcriptome data for the *C. albicans* GI colonization and the translocated infection group (Figure 4E), we investigated the correlations among four distinct bacterial genera, two unique metabolites, and two genes. The co-occurrence network, reconstructed through Spearman correlation analysis, identified four positively and two negatively correlated pairs, underscoring the intricate interactions between the microbiome and the host. For instance, *Dubosiella* sp000403415 and *Mucispirillum schaedleri* were linked to the production of D-erythro-imidazolylglycerol phosphate, which corresponded with the reduced expression of PD-1 in colon tissues of mice subjected to *C. albicans* colonization. Additionally, *Mucispirillum schaedleri* appeared to influence the expression of Ubd through serotonin modulation. These network changes suggest a mechanism by which *C. albicans* colonization in the intestinal tissue could progress to subsequent translocated infection.

### 3.6 *Candida albicans* invasive infection disrupted intestinal barrier and induced immune cell infiltration as well as inflammatory factor expression

**Figure 5:**
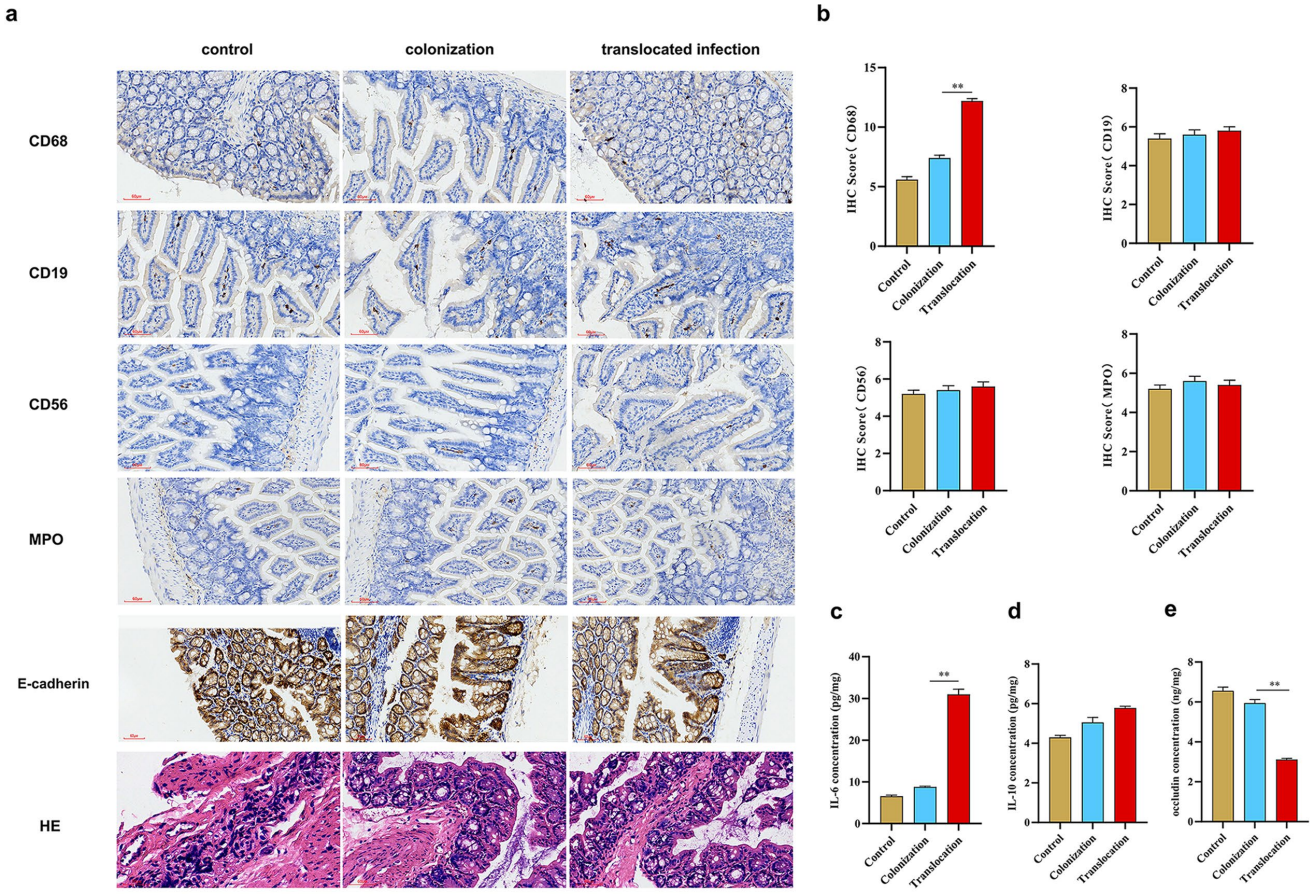
*C. albicans* translocated and invasive infections affect the intestinal barrier, immune cell infiltration and inflammatory factor expression. To assess the characteristics of immune cell infiltration and intestinal barrier integrity in colon tissues subjected to *C. albicans* colonization and subsequent translocated infection, immunohistochemical staining was conducted on mice across three groups. Additionally, we examined the impact of *C. albicans* colonization and translocated infection on the expression of occludin and inflammatory factors, specifically IL-6 and IL-10. **(A)** Immunohistochemical staining and HE staining to evaluate CD56^+^ lymphocytes, CD19^+^lymphocytes, MPO^+^ neutrophil, CD68^+^macrophages, E-cadherin expression and the histopathological injury (scale bar is 60 μm) in three groups. **(B)** Statistics of expression of infiltrated immune cells in three groups. **(C–E)** Statistics of expression of inflammatory factor (IL-6 and IL-10) and occludin in three groups (*p < 0.05, **p < 0.001).

To assess immune cell infiltration in colon tissues following *C. albicans* colonization and translocation infection, immunohistochemical staining for CD68, CD19, CD56, and MPO was performed on samples from each group. Figure 5 reveals that CD56^+^ lymphocytes, CD19^+^ lymphocytes, MPO^+^ neutrophils, and CD68^+^ macrophages were present across all groups. Notably, an increased presence of CD68^+^ macrophages was detected in colon tissues of mice with *C. albicans* colonization and subsequent translocation infection. Conversely, translocation infection did not markedly increase the counts of CD56^+^ lymphocytes, CD19^+^ lymphocytes, or MPO^+^ neutrophils in the colonized mice (Figure 5).

This study also examined the impact of *C. albicans* GI colonization and subsequent translocation infection on the expression of tight junction (TJ) proteins in the colon. Figure 5 indicates a marginal reduction in occludin levels due to GI colonization, whereas mice subjected to subsequent translocation infection exhibited significantly reduced occludin concentrations. Furthermore, we analyzed the effect of *C. albicans* colonization and translocation infection on inflammatory cytokine expression, focusing on IL-6 and IL-10 levels. Post-colonization challenge, IL-6 expression mildly increased. In contrast, IL-6 levels were significantly elevated in colon tissues from mice treated for translocated infection, while IL-10 expression remained unchanged across all groups.

## 4 Discussion

In this study, we observed that all-cause mortality rate in the hospital for *C. albicans* bloodstream infections was approximately 32%. Despite the effectiveness of antifungal agents against fungal pathogens, mortality rates from invasive fungal infections remain high. Our study specifically excluded patients who with HIV infection, undergone stem cell, bone marrow transplantation or receiving high-dose immunosuppressive therapies, respectively. This exclusion criterion leads to observed mortality rate of 32% in our cohort, which is lower than the 40% mortality rate reported in other studies (Bromuro et al., 2024; Drummond et al., 2022). Factors contributing to this mortality rate include extended hospital stays, deteriorated general health, comorbid conditions, critically ill patient status, and limited options for antimicrobial treatment. Crucially, the progression from *C. albicans* rectal carriage to clinical infection was predominantly observed in patients with lower serum protein levels. Hence, clinicians should be trained to take extra precautions with patients exhibiting *C. albicans* rectal carriage and reduced serum protein levels.

*Candida albicans* occupies ecological niches on the human skin and gastrointestinal tract. In immune-compromised hosts, prior colonization by *C. albicans* precipitates opportunistic invasive infections of the skin and mucosa or life-threatening bloodstream infections. A recent study (Mesquida et al., 2023), utilizing species-specific microsatellite markers, compared *C. albicans* genotypes from rectal swabs of patients with pre-collected and analyzed blood samples resulting in identifying the gastrointestinal tract as a potential reservoir for *Candida* spp. capable of triggering candidemia. Both our animal studies and clinical trial have reconfirmed the theory that gastrointestinal colonization by pathogens is a fundamental prerequisite for the onset of translocated infections.

This study explore the direct investigation into whether changes in microbial ecology and bacterial metabolites influence host gene expression, facilitating the progression of *C. albicans* colonization in intestinal tissue to subsequent translocated infection. Transcriptome sequencing and real-time quantitative PCR validation revealed down-regulation of PD-1 and Ubd in colon tissues from mice with *C. albicans* translocated infection compared to GI colonization group. T cell activation is intricately controlled by co-stimulatory molecules to prevent excessive T cell activity. The study (Li et al., 2024) establishes PhagoPL as a useful approach to quantifying the collection of proteins enriched in phagosomes during host–microorganism interactions, exemplified by identifying PD-L1 as a receptor that binds to fungi. It is rapidly induced on T cells upon activation, interacting with its ligands PD-L1 and PD-L2 to deliver a suppressive signal, leading to T cell dysfunction, apoptosis, and ineffectiveness (Chen et al., 2023). Furthermore, the decreased PD-1 expression in colon tissues may contribute to functional activation in response to *C. albicans* colonization. While previous research has noted PD-1 expression changes in T cells and antigen-presenting cells in bacterial sepsis (Zhong et al., 2023) or Candida bloodstream infections (Spec et al., 2016; Wurster et al., 2022), no studies have yet linked PD-1 expression alterations in colon tissues to the transition from *C. albicans* colonization to translocated infection. These findings highlight a complex immune landscape, often associated with increased mortality despite antifungal treatment and persistent immune deficiencies in survivors.

Our study revealed that the interaction between down-regulated *Dubosiella sp000403415* and up-regulated *Mucispirillum schaedleri* within the altered microbial ecosystem likely contributed to a compromised immune status. This state is characterized by the activation of immune checkpoint pathways, potentially mediated by specific bacterial metabolites such as D-erythro-imidazolylglycerol phosphate (HisF). Notably, the presence of *Mucispirillum* was significantly increased in bacterial communities affected by translocated infection. To date, *Mucispirillum* is recognized as the sole genus from the *Deferribacteraceae* family residing in the vertebrate gastrointestinal tract, primarily utilizing mono-saccharides, amino acids, or SCFAs for energy metabolism and has been linked to GI inflammation in numerous studies (Shen et al., 2022; Herp et al., 2021; Herp et al., 2019). Our findings also revealed a correlation between *C. albicans* translocated infection and increased intestinal mucosa inflammation. This suggests that a decrease in beneficial bacteria, along with an overgrowth of *Mucispirillum* spp. and *C. albicans*, may induce inflammation in the GI tract. High level of inflammation is influenced by several factors, particularly the involvement of innate immune cells, such as CD68^+^ macrophages. Furthermore, Treg cells may exacerbate GI tract inflammation via the expression of IL-10. Interestingly, Mucispirillum’s ability to respire nitrate, a compound that proliferates during inflammatory implies a complex interaction with the host’s immune response, leading to exacerbated inflammation. Notably, impaired neutrophil recruitment and bacterial clearance result in elevated *Mucispirillum* levels (Caruso et al., 2019). In the context of *C. albicans* translocated infection, we noted a correlation between the increased *Mucispirillum* and more severe inflammation, indicating a nuanced regulation of inflammatory response at the site. However, it remains ambiguous whether the elevated levels of *Mucispirillum* are cause or effect of immune cell infiltration. Intriguingly, we observed a notable decrease in the relative abundance of *Dubosiella* in samples from mice with *C. albicans* GI colonization, particularly in models of subsequent translocated infection. Recent research (Zhang et al., 2024) highlighted *Dubosiella newyorkensis* in modulating Treg/Th17 responses and enhancing mucosal barrier integrity through SCFA production. *Dubosiella newyorkensis* acts as a probiotic immune-modulator, promoting immune tolerance in dendritic cells by activating indoleamine-2,3-dioxygenase 1 via the aryl hydrocarbon receptor, driving Trp catabolism toward the kynurenine pathway. In this study, in contrast to *Mucispirillum* profile, *Dubosiella* likely exerted a negative influence on the complex immune landscape via HisF. Additionally, emerging evidence suggests that *C. albicans* in the gut actively contributes to *Clostridium difficile* infection through modulation of the gut-brain axis (Henderickx et al., 2023; Markey et al., 2018). Therefore, the serotonin indicator was more likely altered by elevated *C. albicans*, suggesting binary regulatory role of *C. albicans* in modulating *Mucispirillum* abundance, virulence and host serotonin metabolism. Seelbinder et al. (2020) reported that SCFAs and *C. albicans* levels were also identified in stool from healthy human subjects who under-went antibiotic treatment, and, *in vitro*, both acetate and propionate inhibited *C. albicans* growth and reduced its ability to damage epithelial cells. According to results listed above, we concluded the essential role of *Mucispirillum* and *Dubosiella* in establishing immune integrity, while perturbation leads to compromised immune responses attributing to high mortality rate. The alteration in *Dubosiella* and SCFA levels, alongside increased *Mucispirillum* and associated metabolites (HisF and serotonin), could serve as indicators in *C. albicans* rectal colonized patients with lower serum protein levels. Our results underline the necessity of recognizing and addressing the asymptomatic carrier state to prevent *C. albicans* infection severity. Furthermore, strategies aimed at enhancing host immunity could be crucial for improving survival rates, particularly among patients with rectal colonization by *C. albicans* who exhibit lower serum protein levels, reduced SCFAs levels, diminished *Dubosiella* abundance, and increased *Mucispirillum* loads.

Numerous studies (Martin-Gallausiaux et al., 2021; Singh et al., 2023) have shown that SCFAs restore barrier functionality by positively regulating the expression of TJ proteins, leading to enhanced transepithelial electrical resistance. Therefore, we infer that reduced SCFA levels may be linked to lower TJ protein expression level in mice colonized by *C. albicans*. Importantly, this reduction in SCFAs aligns with a decreased presence of SCFA-producing microbiota, including *Dubosiella*, *Faecalibaculum*, and *Bifidobacterium*. Meanwhile, the inverse relationship between SCFA levels, *Dubosiella* and *Desulfitobacterium_A* loads during the progression from *C. albicans* colonization toward invasive infection is not well understood (Wang et al., 2023; Krishnamurthy et al., 2023). Future research should aim to elucidate the paradoxical mechanisms underlying this association. Increased *Mucispirillum* levels and severe inflammation are also associated to SCFA levels decrease and serotonin disturbance. This study additionally revealed that beyond the well-recognized roles of SCFAs and serotonin (Stasi et al., 2019; Grondin and Khan, 2023), D-erythro-imidazolylglycerol phosphate (HisF) may significantly contribute to microbiota balance turn to influence the immunity. However, the specific mechanism of how HisF interacts with the host’s intestinal epithelia and potential intestinal translocation remains to be elucidated.

On the other hand, we noted a significant increase in *Adlercreutzia* abundance alongside a marked decrease in *Faecalibaculum* levels when subjected to *C. albicans* challenge in GI colonization models. Furthermore, multi-omics analysis across the microbiome-metabolite-host axis in mice with *C. albicans* colonization indicated that artemisinin and huperzine B may contribute to the *C. albicans* colonization. However, the mechanisms by which artemisinin and huperzine B influence host gene expression remain to be clarified. In our study, a majority of bacteria maintained levels comparable to those in control mice in response to *C. albicans* GI colonization. Similarly, metabolomics analysis revealed that the intestinal metabolome pattern in colonization models resembled that of the control group. Consequently, these findings collectively suggest that antifungal treatment may not be necessary for patients exhibiting nonpathogenic colonization by *C. albicans*, especially in immune-competent individuals without diarrhea or high-risk factors.

## 5 Conclusion

In summary, prior gastrointestinal colonization by *C. albicans* is a prerequisite to establish opportunistic invasive infections or life-threatening bloodstream infections. The reduced expression of PD-1 in colon tissues may contribute to the transition from colonized *C. albicans* to subsequent translocated infection. The indicator features of decreased Dubosiella abundance and SCFA levels, coupled with increased Mucispirillum and D-erythro-imidazolylglycerol phosphate, are likely linked to the development of translocated invasive infection in hosts colonized rectally by *C. albicans* with lower serum protein levels. Furthermore, SCFAs, D-erythro-imidazolylglycerol phosphate, and serotonin seems play a multifaceted role in modulating immune cell infiltration, inflammatory response, and intestinal barrier function. Given the similarity in intestinal bacterial communities and metabolomic profiles, antifungal treatment may not be necessary for patients exhibiting nonpathogenic colonization by *C. albicans*. While multi-omics analyses and indicator features for potential infections in hosts colonized by *C. albicans* have been investigated, the detailed interactions between *C. albicans*, indicator bacteria, and underlying host mechanisms of intestinal translocation remain to be clarified. Further research is required to unravel this complex interplay with microbe-microbe and microbe-host interactions.

## Data Availability

The raw data from the 16S rRNA gene sequence and transcriptomic experiment have been deposited respectively in the SRA database of NCBI under SRA accession no. PRJNA1081161 and PRJNA1087063 (https://www.ncbi.nlm.nih.gov/guide/). The raw data from metabolomic experiment can be found at Metabolights under accession number MTBLS9750 (https://www.ebi.ac.uk/metabolights).

## Footnotes

^1^ https://www.kegg.jp/kegg/pathway.html

## Supplementary material

SUPPLEMENTARY FIGURE 1 Evaluation of *C. albicans* colonization of the gastrointestinal tract and subsequent infection in immune-compromised mice. **(A,B)** *C. albicans* quantitative culture of colonial contents **(A)** and kidney **(B)** collected from *C. albicans* colonization of the gastrointestinal tract and subsequent infection mice with immunosuppression. **(C,D)** Relative mRNA expressions of 18S gene in the colonial contents **(C)** and kidney **(D)** harvested from *C. albicans* GI colonization and subsequently translocated infection mice. **(E)** Direct staining was used to detect *C. albicans* in blood samples of translocated infection mice. **(F)** *C. albicans* immunofluorescence staining in kidney tissues harvested from *C. albicans* GI colonization and subsequently translocated infection mice (***p* < 0.001).
