# Supplementary figures and images for: Multi-omics analysis reveals indicator features of microbe-host interactions during Candida albicans colonization and subsequent infection
Source: Front Microbiol. 2024 Nov 27;15:1476429. doi: 10.3389/fmicb.2024.1476429 (PMC11632224; doi:10.3389/fmicb.2024.1476429)

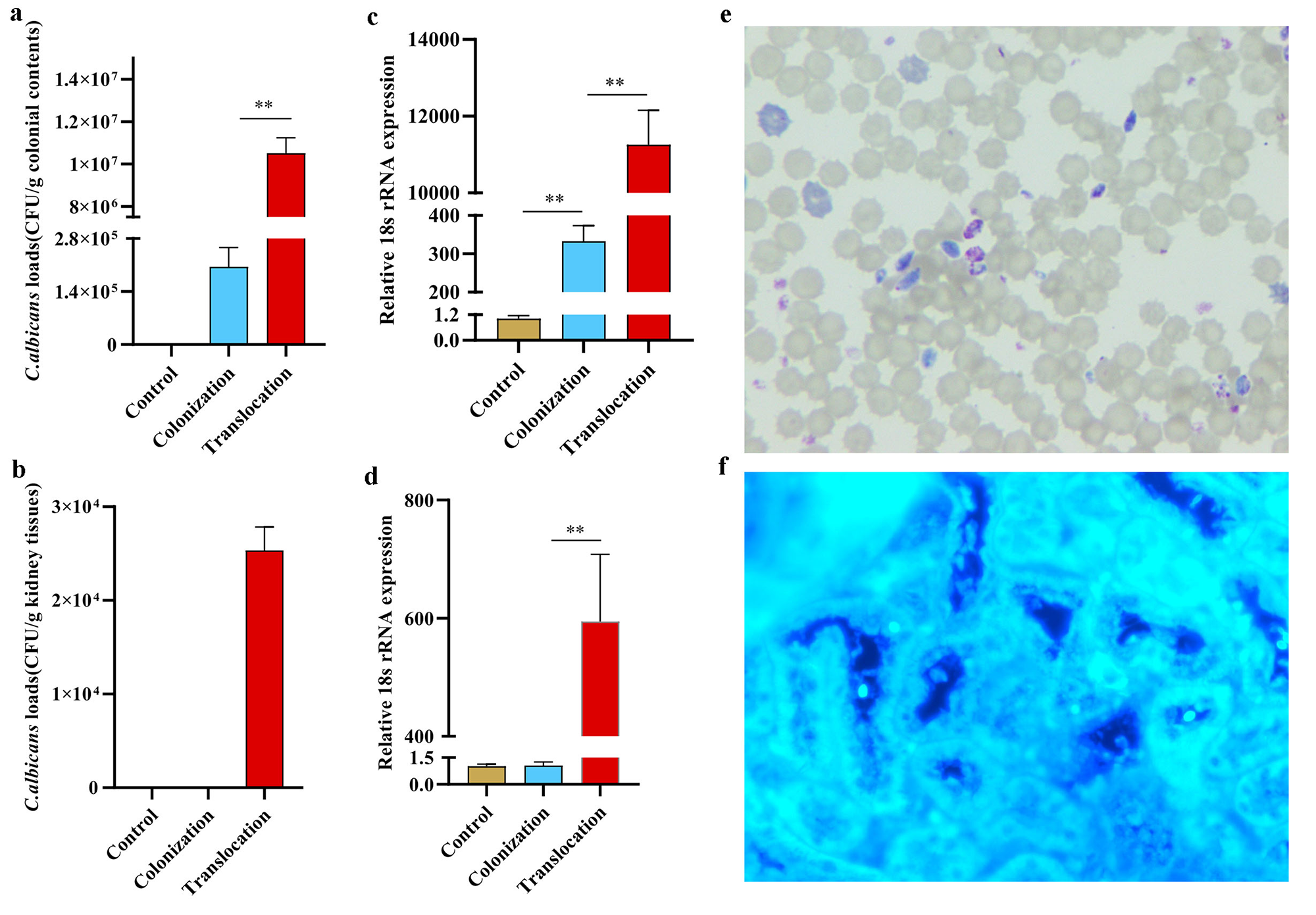

Supplement: SUPPLEMENTARY FIGURE 1 — Evaluation of C. albicans colonization of the gastrointestinal tract and subsequent infection in immune-compromised mice. (A,B) C. albicans quantitative culture of colonial contents (A) and kidney (B) collected from C. albicans colonization of the gastrointestinal tract and subsequent infection mice with immunosuppression. (C,D) Relative mRNA expressions of 18S gene in the colonial contents (C) and kidney (D) harvested from C. albicans GI colonization and subsequently translocated infection mice. (E) Direct staining was used to detect C. albicans in blood samples of translocated infection mice. (F) C. albicans immunofluorescence staining in kidney tissues harvested from C. albicans GI colonization and subsequently translocated infection mice (**p < 0.001). [file Image_1.TIF]
